# Tricuspid endocarditis secondary to Salmonella typhi infection: a case report

**DOI:** 10.1093/ehjcr/ytag615

**Published:** 2026-08-20

**Authors:** Mouad Lamtai, Idriss Allalat, Mehdi Moujahid, Nadia Fellat

**Affiliations:** Cardiology A Department, Ibn Sina University Hospital/Mohammed V University Rabat, 10105 Rabat, Morocco; Cardiology A Department, Ibn Sina University Hospital/Mohammed V University Rabat, 10105 Rabat, Morocco; Cardiology A Department, Ibn Sina University Hospital/Mohammed V University Rabat, 10105 Rabat, Morocco; Cardiology A Department, Ibn Sina University Hospital/Mohammed V University Rabat, 10105 Rabat, Morocco

**Keywords:** Infective endocarditis, Pulmonary infarcts, Salmonella typhi, Transthoracic echocardiography, The chest CT scan, Case report

## Case summary

We present a rare case of Salmonella typhi causing tricuspid valve endocarditis in a 60-year-old woman, complicated by septic pulmonary embolism and pulmonary infarctions. Salmonella typhi endocarditis is uncommon and typically affects left-sided valves; right-sided involvement with septic pulmonary embolic complications is rare and poorly documented. The patient was managed with antibiotic therapy, highlighting an unusual clinical presentation and a non-surgical management strategy.

## Case presentation

A 60-year-old woman with a history of untreated hyperthyroidism presented to the emergency department with 20 days of prolonged fever, diarrhoea, abdominal pain, and class III NYHA dyspnoea.

The physical exam showed a hypoxic patient with an oxygen level of 88%, blood pressure of 100/56 mmHg, and heart rate of 110 b.p.m. Cardiovascular exam showed a systolic murmur in the fourth intercostal space and basal crackles on auscultation.

**Figure 1:**
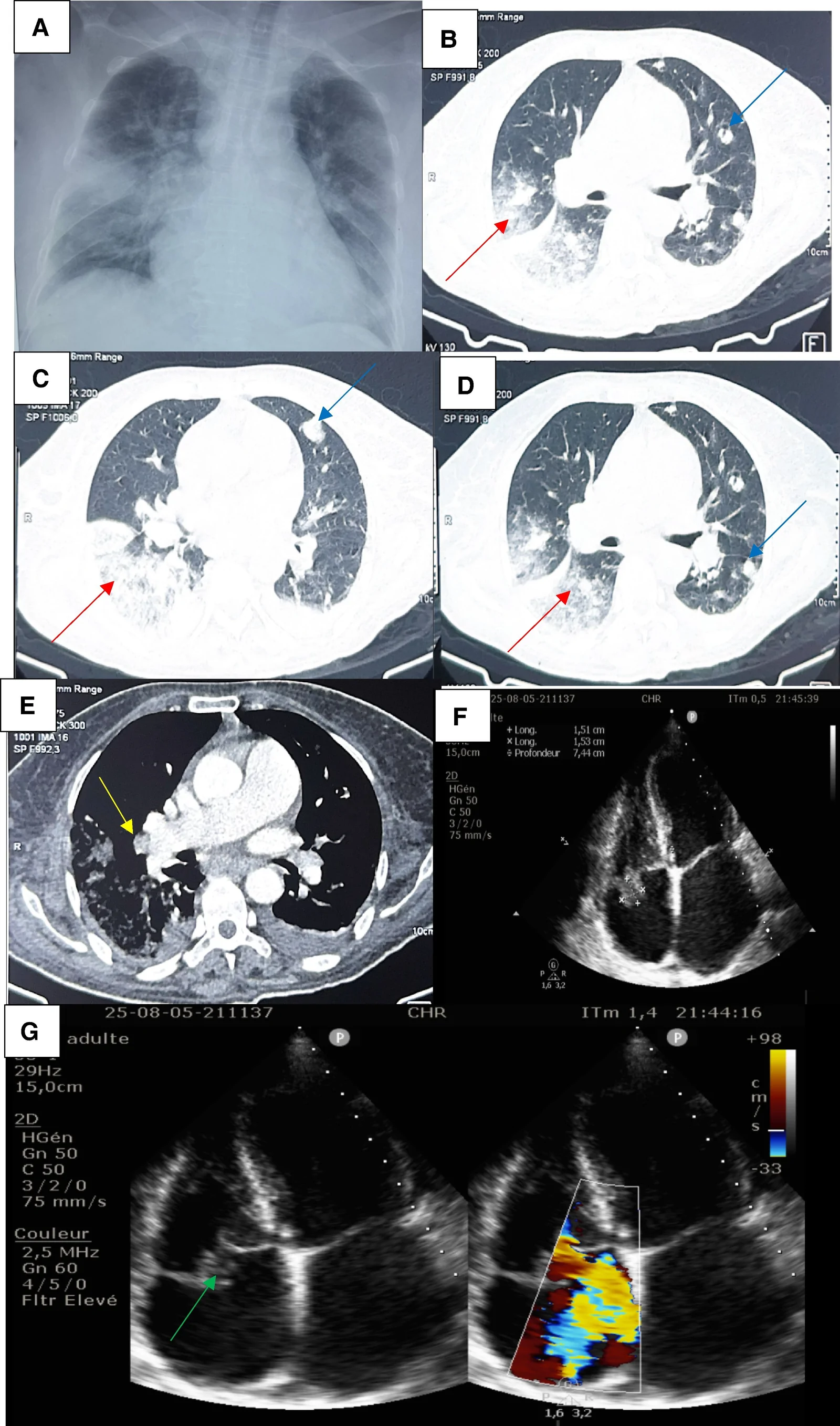
(*A*) Chest X-ray showed cardiomegaly, straightening of the pulmonary arch, and nodular pulmonary infiltrates in both lungs with a localized right pulmonary opacity. (*B–E*) The chest CT scan showed multiple consolidative areas with ground-glass opacities (red arrow), multiple bilateral pulmonary nodules (blue arrow)—most of which show a feeding vessel sign—a segmental septic pulmonary embolism (yellow arrow), and bilateral pleural effusion. (*F, G*) Transthoracic echocardiography revealed a mobile structure measuring 15 × 15 mm, adherent to the posterior leaflet of the tricuspid valve, causing leaflet prolapse (green arrow) and resulting in eccentric moderate tricuspid regurgitation.

Chest X-ray showed cardiomegaly, nodular pulmonary infiltrates with localized right pulmonary opacity (*Figure 1A*). The chest CT scan showed multiple consolidative areas, ground-glass opacities, multiple pulmonary nodules—most of which show a feeding vessel sign—segmental septic pulmonary embolism, and bilateral pleural effusion (*Figure 1B–1D*).

Transthoracic echocardiography revealed a mobile structure measuring 15 × 15 mm, adherent to the posterior leaflet of the tricuspid valve, causing leaflet prolapse and resulting in eccentric moderate tricuspid regurgitation with maximal velocity of 3.43 m/s (*Figure 1F–1G*).

Laboratory tests showed bicytopenia with elevated infection and inflammatory markers. Two blood cultures confirmed the presence of Salmonella typhi. The Widal test was also positive, although its limited specificity is acknowledged.

The diagnosis of endocarditis was established according to the 2023 ESC Guidelines for the management of infective endocarditis.^1^ In-hospital treatment consisted of intravenous ceftriaxone 4 g/24 h combined with ciprofloxacin 400 mg every 12 h according to antimicrobial susceptibility testing of the isolated Salmonella typhi. After 6 weeks of therapy, blood cultures became sterile, the patient was afebrile, and the infection was controlled with clinical stabilization. Follow-up echocardiography demonstrated stable tricuspid regurgitation with no significant change in vegetation size. The Heart Team decided not to intervene, as there was no persistent infection, recurrent pulmonary embolism, or severe tricuspid regurgitation.

Salmonella typhi is a rare cause of endocarditis, with limited case reports. The mitral and aortic valves are the most affected. Antibiotic therapy is the mainstay of treatment, showing favourable results.^2^ Cardiac surgery was reserved for specific indications such as severe valvular insufficiency, extensive valvular destruction, or prosthetic abscess.^3^

## Data Availability

Data sharing is not applicable as no data sets were generated or analysed for this case report.

## Notes

**Consent:** Informed consent was obtained from the patient in accordance with COPE guidelines.
